# Evaluation of immune response after moderate and overtraining exercise in wistar rat

**Published:** 2014-01

**Authors:** Zahra Gholamnezhad, Mohammad Hossein Boskabady, Mahmoud Hosseini, Mojtaba Sankian, Abolfazl Khajavi Rad

**Affiliations:** 1 Neurogenic Inflammation Research Centre and Department of Physiology, School of Medicine, Mashhad University of Medical Sciences, Mashhad, Iran; 2 Neurocognitive Research Centre, School of Medicine, Mashhad University of Medical Sciences, Mashhad, Iran; 3 Immunology Research Center, School of Medicine, Mashhad University of Medical Sciences, Mashhad, Iran

**Keywords:** Immune system, Moderate exercise, Overtraining exercise, Rat, Th1, Th2

## Abstract

***Objective(s):*** The effect of prolonged overtraining on cytokine kinetics was compared with moderate exercise in the present study.

***Materials and Methods:*** Male Wistar rats were randomly divided into control sedentary (C), moderate trained (MT), (V=20 m/min, 30 min/day for 6 days a week, 8 weeks), overtrained (OT) (V=25 m/min, 60min/day for 6 days a week, 11 weeks) and recovered overtrained (OR) (OT plus 2 weeks recovery) groups, (n=6 for each group). Immediately, 24 hr and 2 weeks (in OR) after last bout of exercise blood samples were obtained. The plasma concentrations of TNFα, IL-6, IL-10, IL-4 and IFN were measured by ELISA method.

***Results:*** Immediately after last bout of exercise the following findings were observed; IL-6, IL-10 and TNFα concentrations increased in OT and OR groups compared with control (*P*<0.05–*P*<0.001). Serum level of IL-4 decreased (*P*<0.01) but IFN increased (*P*<0.05) in MT group vs. control. In addition, circulatory levels of TNFα, IL-6, IL-10 and IL-4 were higher but the IFN concentrations were lower in OT and OR groups than MT group (*P*<0.05-*P*<0.01). The IFN-γ/IL4 ratio was significantly increased in MT (*P*<0.01) while it decreased in OT group. There were not statistical differences in TNFα, IL-6, and IFN levels between different time intervals after exercise in MT, OT and OR groups.

***Conclusion:*** These data confirm a positive effect of moderate exercise on immune function and a decrease in susceptibility to viral infection by inducing Th1 cytokine profile shift. However, prolonged and overtraining exercise causes numerous changes in immunity that possibly reflects physiological stress and immune suppression.

## Introduction

Changes in lifestyle and physical inactivity lead to increased incidence of many chronic non-communi-cable diseases which are major causes of death (36 million each year) and health problems in the world (1–3). Physical inactivity, as does smoking, increases risk for cancer, cardiovascular and chronic lung diseases and diabetes by 20%–30%, and shortens lifespan by 3–5 years (1). On the other hand regular exercise, like a miracle drug, has many beneficial effects on the body and protects against those diseases (4–6). The key recommendation of the recently published World Health Organization guidelines regarding physical activity is "adults should do at least 150 min a week of moderate intensity, or 75 min a week of vigorous intensity aerobic physical activity, or an equivalent combination of moderate and vigorous intensity aerobic activity" (7, 8). However, the research report recommends that future investigations need to evaluate the effects of physical activity intensity at fixed energy expenditure doses (9). This is due to the effects of exercise on the physiologic parameter which depends upon several factors, such as the frequency of each bout and the total duration of the exercise protocol (10).

Although new studies have shown that the site of cytokine production and action are beyond immune system; still they have been considered as secreted proteins which regulate all aspects of innate and adaptive immunity (11). There is a dynamic equi-librium between pro and anti-inflammatory cytokines. The time course of cytokine release, the local cytokine milieu, the existence of stimulating 

and inhibiting factors and their receptor densities are determinants of the net cytokine effect (12). T cell cytokines have a pivotal role in the promotion of immune responses against invading pathogens (13). There are two distinct cytokine producing T cell subtypes: CD4^+^ T helper and CD8^+^ T cytotoxic which have been appointed to type 1 or type 2 T lymphocytes based on their profile of cytokine production (14). Type 1 lymphocytes are essential for the cell-mediated immune and defense against intracellular pathogens by producing interferon γ (IFNγ), interleukin 2 (IL-2) and tumor necrosis factor-β cytokines. Whereas, type 2 lymphocytes produce cytokines including IL-4, IL-5, IL-6, IL-10, and IL-13 are responsible for defense against extracellular pathogens by the development of humoral immunity (15,16). These two classes of cytokines have cross-regulatory signaling; for example IL-4 and IL-10 secretion causes the inhibition of Th1-type immune responses by down-regulating macrophage-derived IL-12 production. Also IFNγ changes the balance of Th1/Th2 by suppressing the Th2-type immune responses (17).

According to hormesis theory, the responses of biological systems to stressors may be explained by the U-shaped curve. The two endpoints of this curve are inactivity and overtraining, and both of these result in decreased physiological function (18, 19). Stress may weaken host defense against external pathogens or stimuli and internal tumor develop-ment by impairing immune responses such as antibody production, natural killer (NK) activity, and lymphocyte responses to mitogens (20). It has been reported that moderate or intermittent exercise enhances immune function but prolonged and sever exercise cause numerous changes in immunity, which possibly reflects physiological stress and suppression (19, 21). Athletes tolerating more intense levels of training may be at increased risk of upper respiratory tract infection (URTI) during periods of sever exercise and for the few weeks after race events (22). Interestingly, most of the studies used applied voluntary exercise, even though the effects of enforced physical exercise, especially with different loads, are unclear. There is little information regarding whether regular exercise above a certain intensity or duration could be harmful. It would be of interest to identify the optimum exercise loading which could improve certain physiological aspects, including immune function. According to the current findings of adaptation to physical exercise, until an overtraining syndrome appears, regular exercise has beneficial effects (23). However, with overtraining, which is still a poorly understood process, the homeostatic balance involving a wide range of hormonal, metabolic, and immunologic factors is altered (24).

Many studies had evaluated post exercise immunomodulation in human, especially Olympic and marathon race athletes. However, the study of forced sever exercise and overtraining syndrome in human has clear ethical limits that are unquestioned in the literature (21, 25, 26). Different kinds of exercise may have various effects on immune parameters based on the nature, intensity and time delay between exercise bouts and immune parameter. In addition, it is not clear what part of this multifactorial stress (psychological or physical) in those human studies effects components of the immune system (27). Therefore, more evidence is needed to explain the nature and clinical feature of this immunomodulation. Consequently, in the pre-sent study we examined the effect of moderate and sever treadmill running on plasma concentrations of TNFα, IL-6, IL-10, IL-4 and IFN cytokines immedia-tely, 24 hr and 2 weeks after the last bout of exercise.

## Materials and Methods

***Animals***

Thirty adult (6–8 weeks old) male Wistar rats, (Animal House of School of Medicine, Mashhad University of Medical Sciences, Mashhad, Iran) weighing 150–200 g were used. Animals were housed under environmentally controlled conditions (12 hr light/dark cycle; 22–24°C) and food and water were available *ad libitum* throughout the experiment. Animals were allowed to adjust to new condition for two weeks. The protocols used conformed to guidelines of animal studies and were approved by the committee on the ethics of animal experiments in Mashhad University of Medical Sciences. 

***Training protocol***

A motorized treadmill with 4 individual lanes was used. A shock grid at the back of the treadmill provided a mild shock (0.5 mA, 1 HZ) if the rat's pace went below the treadmill rate. The animals under-took a one week familiarizing period prior to the beginning of the experiments. They were placed on the treadmill 10 min/day for 5 days at a speed of 12 m/min at 0% degree inclination. Then they were scored 1–5 depending on running quality; rats that ran voluntarily with a mean rating of 3 or higher (n=24) were separated from those which refused (n=6) and those with a 3 or higher score were chosen for the study. This procedure was used to exclude possible different levels of stress between animals (25).

Twenty four rats were randomly divided into four equal groups including: control sedentary (C), moderate trained (MT), overtrained (OT) and recov-ered overtrained (OR). 

The animals of the control group were handled and placed on the treadmill with the aim of experiencing the stress of treadmill environment. Exercised groups undertook a progressive load training 6 days a week to enhance cardiorespiratory fitness and a 5 minute warm up and cool down were included in each session. MT group underwent 8 weeks exercise at a speed of 15 m/min for 20 min, 6 days/week but the intensity of exercise was increased to 20 m/min for 30 min at the onset of the second week (28). OT and OR groups were submitted into the 3 phase program. In the first 4 weeks (phase I) training speed increased from 15 to 25 m/min and training time from 20 min to 60 min. In the second 4 weeks (phase II) training load was kept constant. During last 3 weeks (phase III) running intensity and training duration remained unchanged but recovery time between training sessions was reduced (from 24 hr to 4, 3 and 2 hr). The OR group had a 2 weeks recovery period after the last exercise session (25). The training program was evaluated by a perform-ance test at the end of each phase.

**Figure 1:**
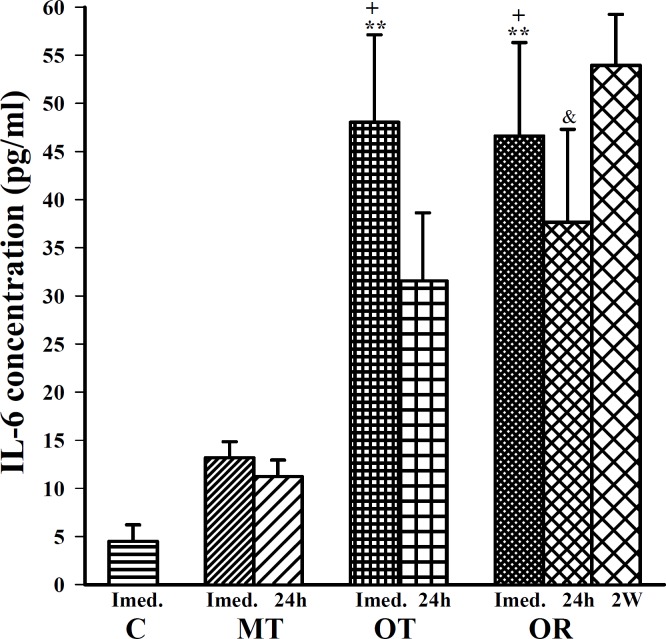
The levels of serum IL-6 in control sedentary rats (C), moderate trained (MT), overtrained (OT), recovered overtrained (OR), (for each group, n=6).

Statistical differences between control and other groups (immediately after last bout of exercise): **; *P* <0.01.

Statistical differences between MT* vs* OT and OR groups (immediately after last bout of exercise): ^+^; *P* <0.05.

Statistical differences between MT* vs* OR group (24 hr after last bout of exercise): ^&^; *P* <0.05.

There were not statistical differences between different times in MT, OT and OR groups

***Sample collection***

At the end of the study, animals were anesthetized with diethyl ether; peripheral blood was collected from the retro-orbital sinus in the control group, immediately and 24 hr after the last session of exercise in MT and OT groups and immediately, 24 hr and 2 weeks after the last session of exercise in OR group. After allowing blood to clot on ice, serum samples were separated by centrifuging at 3000 rpm for 10 min. Serum was collected and stored at -20 C for cytokine analysis.

***Cytokine assays***

Cytokine determination was performed with commercially available platinum ELISA kits (Bender Med system, Austria) according to manufacturer’s instruction. They were carefully checked for specify, sensitivity and reliability. Plasma concentration of IL-4, IL-6, IL-10, TNFα and IFN were measured using rat ELISA kits: BMS628, BMS625, BMS629, BMS622 and BMS621 respectively. The absorbance was measured using a spectrophotometer and a microplate reader (Biotek, USA); concentration of each cytokine was calculated by a comparison curve established in the same measurement using prism 5 Graphpad. Each cytokine assay was performed in duplicate each time.

**Figure 2:**
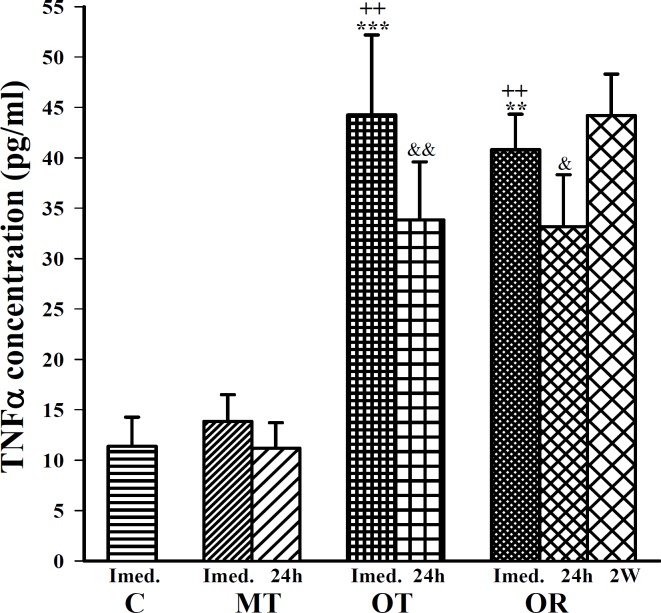
The levels of serum TNF  in control sedentary rats (C), moderate trained (MT), overtrained (OT), recovered overtrained (OR), (for each group, n=6).

Statistical differences between control and other groups (immediately after last bout of exercise): **; *P* <0.01, ***; *P* <0.001.

Statistical differences between MT* vs* OT and OR groups (immediately after last bout of exercise): ^++^; *P* <0.01, ^+++^; *P* <0.001.

Statistical differences between MT* vs* OR group (24 hr after last bout of exercise): ^&^; *P* <0.05; ^&&^; *P* <0.01.

There were not statistical differences between different times in MT, OT and OR groups

***Statistical analysis***

The results were presented as means ± SEM. Group-data comparisons were performed using one way analysis of variance (ANOVA) with Tukey-Kramer post-test. The impact of time on each group data was evaluated by paired t-test for MT and OT groups, and by repeated measurement of ANOVA for OR group. Significance was accepted at *P*<0.05.

## Results

***The effect of exercise on serum concentration of cytokines***

**Figure 3:**
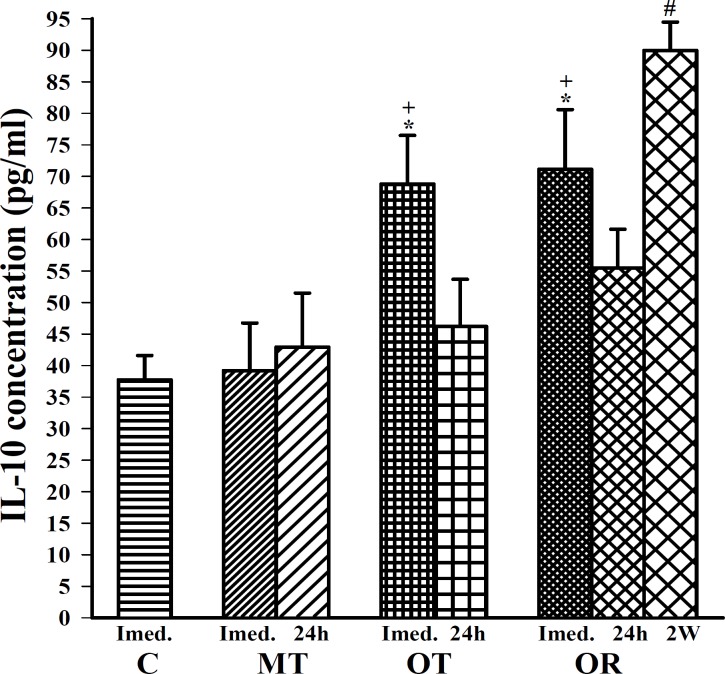
The levels of serum IL-10 in control sedentary rats (C), moderate trained (MT), overtrained (OT), recovered overtrained (OR), (for each group, n=6).

Immediately after last bout of exercise the following finding was observed; IL-6 concentration was significantly increased in OT and OR groups compared with control (F (3, 20)=10.82, *P*<0.01 for both groups), (Figure 1). In addition, TNFα and IL-10 concentrations were significantly increased in OT and OR groups compared with control (F(3, 20) =13.32, *P*<0.001 for TNFα and F(3, 20)=6.02, *P*<0.01 for IL-10 in both groups), (Figure 2 and 3).

**Figure 4:**
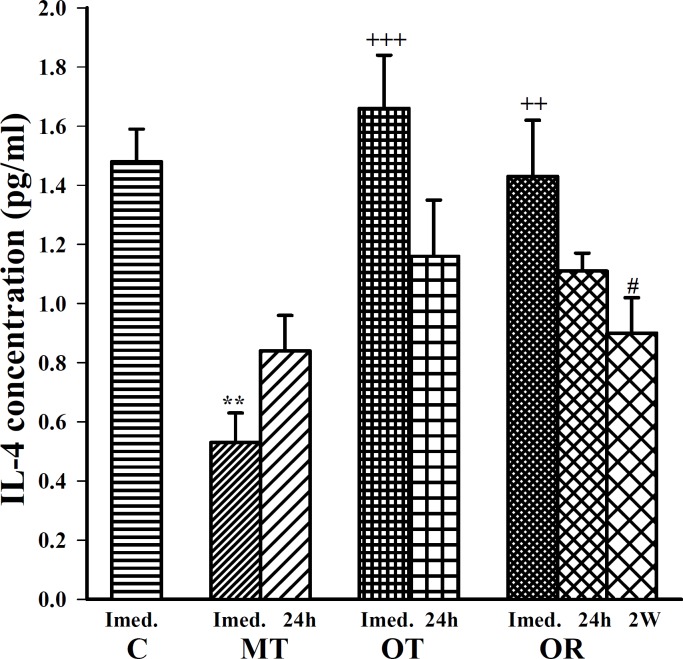
The levels of serum IL-4 in control sedentary rats (C), moderate trained (MT), overtrained (OT), recovered overtrained (OR), (for each group, n=6)

**Figure 5:**
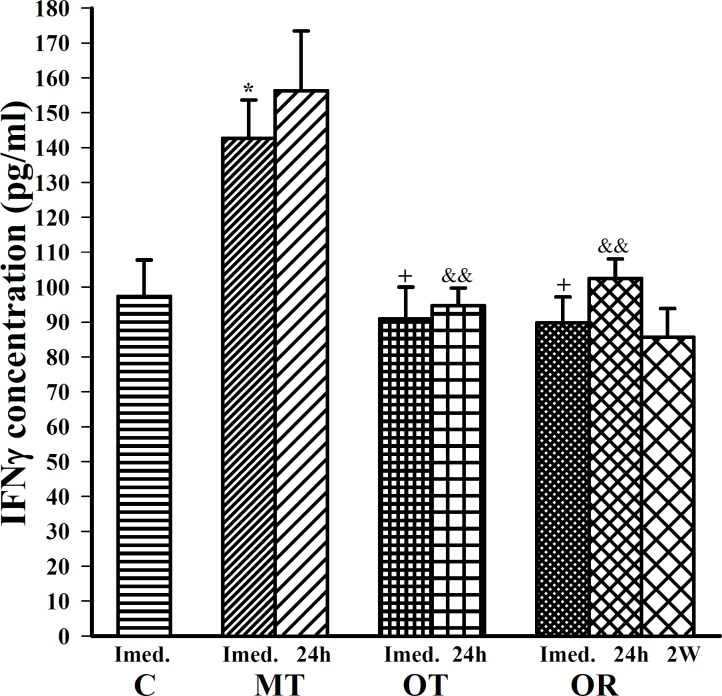
The levels of serum IFN  in control sedentary rats (C), moderate trained (MT), overtrained (OT), recovered overtrained (OR), (for each group, n=6).

Serum level of IL-4 was significantly decreased (F=11.65, *P*<0.001), (Figure 4); serum level of IFN was significantly increased only in MT group compared with control group (F(3,20)=5.44, *P*<0.05), (Figure 5). There were no significant differences in serum levels of IFN and IL-4 in OT and OR groups compared with control (Figure 5).

Statistical differences between control and other groups (immediately after last bout of exercise): *; *P* <0.05.

Statistical differences between MT* vs* OT and OR groups (immediately after last bout of exercise): ^+^; *P* <0.05.

Statistical differences between different times: 24 hr and 2 weeks post exercise: ^#^; *P* <0.05

There were not statistical differences between different times in MT and OT

Statistical differences between control and other groups (immediately after last bout of exercise): **; *P* <0.01.

Statistical differences between between MT* vs* OT and OR groups (immediately after last bout of exercise): ^++^; *P* <0.01, ^+++^; *P* <0.001.

Statistical differences between different times: immediately and 2 weeks post exercise: ^#^; *P*<0.05

There were not statistical differences between different times in MT and OT

Statistical differences between control and other groups (immediately after last bout of exercise): *; *P* <0.05, **; *P* <0.01.

Statistical differences between MT* vs* OT and OR groups (immediately after last bout of exercise): ^+^; *P* <0.05.

Statistical differences between MT* vs* OR group (24 hr after last bout of exercise): ^&&^; *P* <0.01.

There were not statistical differences between different times in MT, OT and OR groups

***The effect of exercise intensity on serum concentration of cytokines***

In this study, immediately after last bout of exercise serum levels of IL-6, TNFα, IL-10 and IL-4 were higher in OT and OR groups than MT group (F(3,20)=10.82, *P*<0.05 for IL-6; F(3,20)=13.32, *P*<0.01 for TNFα; F(3,20)=6.02, *P*<0.05 for IL-10; for both groups and F(3,20)=11.65, *P*<0.001 in OT and *P6*<0.01 in OR group for IL-4), (Figure 1, 2, 3 and 4). However, IFN concentration was lower in OT and OR groups (F(3,20)=5.44, *P*<0.05 in both cases) than MT group (Figure 5).

**Figure 6:**
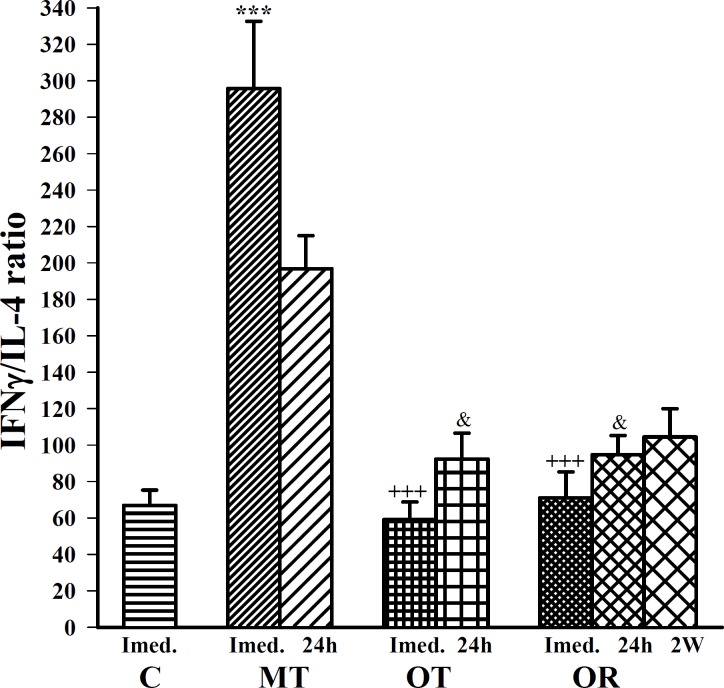
The ratio of serum INF-γ/IL-4 (Th1/Th2 balance) in control sedentary rats (C), moderate trained (MT), overtrained (OT), recovered overtrained (OR), (for each group, n=6).

Statistical differences between control and other groups (immediately after last bout of exercise): ***; *P* <0.001.

Statistical differences between MT* vs* OT and OR groups (immediately after last bout of exercise): ^+++^; *P* <0.001.

Statistical differences between MT* vs* OR group (24 hr after last bout of exercise): ^&^; *P* <0.05.

There were not statistical differences between different times in MT, OT and OR groups

***The acute and chronic effect of exercise on serum concentration of cytokines***

Twenty four hours after last bout of exercise the concentrations of IL-6 in OR was higher than MT group (F(2,15)=3.93, *P*<0.05), (Figure 1). TNFα concentration in OT and OR was also higher than MT group (F(2,15)=7.59, *P*<0.01 for both groups), (Figure 2). In addition, IFN concentration was lower in OT and OR groups than MT group (F (2, 15) =9.65, *P*<0.01 for both groups), (Figure 5). However, there were no significant differences in IL-10 and IL-4 concentrations between OT, OR and MT groups (Figure 3-4). Twenty four hours after last bout of exercise all measured cytokines concentrations were non-significantly decreased compared with immediately after exercise (Figure 1-5). The concentration of IL-10 was significantly higher in 2 weeks than 24 hr after exercise in OR group (F(2,15)=5.11, *P*<0.05), (Figure 3). However, IL-4 concentration was decreased after 2 weeks compared with 24 hr after exercise in this group (F(2,15)=3.89, *P*<0.05 respectively), (Figure 4). IFN concentration did not show remarkable time dependent changes (Figure 5).

***Exercise intensity and Th1/Th2 balance***

Moderate exercise significantly increased (F(3,20)=22.86, *P*<0.001) IFN-γ/IL4 ratio (Th1/Th2 balance) compared with control. However, overtraining caused a non-significant decrement in IFN-γ/IL4 ratio which increased after 2 weeks recovery in comparison to control (Figure 6).

## Discussion

Physiological basis of overtraining as a physical stressor remains poorly understood (25). To our knowledge, this is the first animal study to investigate the cytokine kinetics after prolonged overtraining endurance treadmill exercise in comparison to the moderate intensity one. We used here a standard training protocol for overtraining with various time periods between exercise bouts and rest (25–29). This protocol caused a decline in performance (data not shown), which is the only parameter stated in the literature to be obligatorily associated with overtraining (25, 30). Also we saw marked post exercise changes in circulatory cytokines in the OT group even after 24 hr. Therefore, to examine the reproducibility of our findings in OT group and sustainability of them, the OR group was also formed.

 In this study different sampling times after overtraining showed a chronic increase in pro-inflammatory cytokine TNF and inflammation responsive cytokine IL-6, which remained elevated at least two weeks after recovery. In addition, this load of exercise caused no change in acute IL- 4 (Th2 cytokine) concentration but it was significantly decreased after 24 hr and 2 weeks recovery. However, IL-10 (anti-inflammatory cytokine) concentration showed significant increase immediately post exercise, with a reduction after 24 hr; it increased again after 2 weeks recovery. There was no significant change in IFN (Th1 cytokine) at different time points after exercise. This marked post exercise elevation in IL-6 and IL-10 concentrations is supported by previous observations (11, 27, 31–32). 

It is believed that exercise can induce a primary increase in IL-6, followed by an increase in IL-1ra and IL-10 (11, 26–27, 33). Many probable sources have been proposed for IL-6 elevation after exercise which are related to exercise intensity, duration, the mass of muscle recruited and one's endurance capacity (11, 26, 34). Recent studies have shown that contracting skeletal muscles, but not immune cells, are the main source of IL-6 in the circulation in response to exercise (35). It is shown that IL-6 mRNA is upregulated in contracting skeletal muscles (36-38) and exercise enhances the transcriptional rate of the IL-6 gene (39). Furthermore, adipose tissue may contribute to the exercise-induced augmentation of IL-6 in the circulation but at recovery time (40). It has been proposed that as muscle glycogen level decreases, IL-6 may signal the liver to increase its glucose output and prevent a sever fall in exercise induced glucose concentration which has a lipolytic effect (38, 41-44). 

Previous studies most of which had been performed after marathon races or used the human endurance exercise protocol reported controversial findings for serum TNF (35). Ostrowski *et al* reported: "the plasma level of IL-1beta, TNF alpha, sTNF-r1 and sTNF-r2 peaked in the first hour after exercise (2. 1-, 2.3-, 2.7- and 1.6-fold, respectively)" but in other studies TNF α in serum was not detected and there was no significant increase in TNF-alpha mRNA, in spite of TNF-alpha mRNA detection in resting muscle samples (45-46). It has been demonstrated that a bout of prolonged exercise had no effect on monocytes IL-6 protein expression and TNF or IL-1 production from them (38). However, the exercise protocol (intensity, duration) and other factors like sex, race, and examiners adaptation and even sensitivity and specificity of the test should be considered as main factors of this bias. 

Our data showed that overtraining treadmill running caused prolonged IL-6 elevation. This second increase may be correlated with muscle damage which caused a low-grade inflammation with macrophage and neutrophils penetration to damaged tissue during 6–48 hr post exercise, activating macrophage release of IL-6 as part of an inflammatory response. Moreover, TNF may stimulate IL-6 production (26) and IL-6 in turn, may activate IL-10 and cortisol production (47). It is also proposed that an acute bout of physical activity may cause physiological responses which are very similar in many aspects to those induced by infection, sepsis or trauma (48). Therefore, our findings showed that after prolonged strenuous exercise like acute exercise there is a chronic low systemic inflammation due to TNF and IL-6 elevation which is balanced by increased IL-10 concentration as an anti-inflammatory cytokine.

Type 2 lymphocytes, monocytes and B cells are the main producers of IL-10 and together with IL-4 can inhibit type 1 T cell production (38). In this study we showed a significant reduction in IL-4 and elevation in IFN concentration after moderate exercise, which confirmed the hypothesis that the T1 cell responses are strengthened following moderate intensity exercises (21). The fact that moderate elevation in IL-6 was not accompanied by TNFα and IL-10 changes after moderate exercise may support its anti-inflammatory effects (26). However, the mechanism of this immunomodulatory effect is not completely known to date. It has been suggested that sever overtrained exercise may lead to tissue trauma, which would activate local cells to produce cytokines, stimulating the differentiation of Th2 cells, and also elevate circulating levels of stress hormones, including cortisol and catecholamines, which can inhibit the production of IL-12 (main inducer of Th1 cells) and would up-regulate Th2 lymphocyte responses (49-51). However, in turn, moderate exercise training has been shown to induce down-regulation of the steady state level of 2- adrenergic receptor on macrophages. This effect was associated with decrease in the suppressive effects of catecholamines on IL-12 production, thereby resulting in the up-regulation of Th1 responses (52). It seems that after regular exercise with adequate intensity and duration the body has the capability to cope with the exercise (stressor) and as a result adaptation takes place. This adaptive effect may mediate the health-beneficial effect and play an important role in protection against chronic non-communicable diseases which are associated with low-grade inflammation (53).

## Conclusion

The results of the present study showed that in contrast to moderate or intermittent physical activity, prolonged and overtraining exercise causes numerous changes in immunity that possibly reflect physiological stress and immune suppression, which is in agreement with hormesis theory.

## References

[1] Wen CP, Wu X (2012). Stressing harms of physical inactivity to promote exercise. Lancet.

[2] Lee IM, Shiroma EJ, Lobelo F, Puska P, Blair SN, Katzmarzyk PT (2012). Effect of physical inactivity on major non-communicable diseases worldwide: an analysis of burden of disease and life expectancy. Lancet.

[3] Woodcock J, Franco OH, Orsini N, Roberts I (2011). Non-vigorous physical activity and all-cause mortality: systematic review and meta-analysis of cohort studies. Int J Epidemiol.

[4] Fallah Mohammadi M, Hajizadeh Moghaddam A, Mirkarimpur H (2013). The effects of a moderate exercise program on knee osteoarthritis in male wistar rats. Iran J Basic Med Sci.

[5] Pimlott N (2010). The miracle drug. Can Fam Physician.

[6] Eshraghi-Jazi F, Alaei H, Azizi-Malekabadi H, Gharavi-Naini M, Pilehvarian A, Ciahmard Z (2012). The effect of red grape juice and exercise, and their combination on parkinson’s disease in rats. Avicenna Journal of Phytomedicine.

[7] Organization WH (2010). Global Recommendations on Physical Activity for Health.

[8] Janssen I, Ross R (2012). Vigorous intensity physical activity is related to the metabolic syndrome independent of the physical activity dose. Int J Epidemiol.

[9] (2009). Physical Activity Guidelines Advisory Committee report. To the Secretary of Health and Human Services. Part A: executive summary. Nutr Rev.

[10] Hewitt M, Creel A, Estell K, Davis IC, Schwiebert LM (2009). Acute exercise decreases airway inflammation, but not responsiveness, in an allergic asthma model. Am J Respir Cell Mol Biol.

[11] Gleeson M, Gleeson M (2006). Cytokines and exercise. Immune function in sport and exercise.

[12] Kilciler G, Musabak U, Bagci S, Yesilova Z, Tuzun A, Uygun A (2008). Do the changes in the serum levels of IL-2, IL-4, TNF alpha, and IL-6 reflect the inflammatory activity in the patients with post-ERCP pancreatitis?. Clin Dev Immunol.

[13] Dinarello CA (1998). Interleukin-1, interleukin-1 receptors and interleukin-1 receptor antagonist. Int Rev Immunol.

[14] Mosmann TR, Cherwinski H, Bond MW, Giedlin MA, Coffman RL (1986). Two types of murine helper T cell clone. I. Definition according to profiles of lymphokine activities and secreted proteins. J Immunol.

[15] Abbas AK, Lichtman AH, Pillai S (2012). Immunity to Microbes. Cellular and molecular immunology.

[16] Seder RA, Paul WE (1994). Acquisition of lymphokine-producing phenotype by CD4+ T cells. Annu Rev Immunol.

[17] Kaiko GE, Horvat JC, Beagley KW, Hansbro PM (2008). Immunological decision-making: how does the immune system decide to mount a helper T-cell response?. Immunology.

[18] Radak Z, Chung HY, Koltai E, Taylor AW, Goto S (2008). Exercise, oxidative stress and hormesis. Ageing Res Rev.

[19] Kazeem A, Olubayo A, Ganiyu A (2012). Plasma Nitric Oxide and Acute Phase Proteins after Moderate and Prolonged xercises. Iran J Basic Med Sci.

[20] Oishi K, Nishio N, Konishi K, Shimokawa M, Okuda T, Kuriyama T (2003). Differential effects of physical and psychological stressors on immune functions of rats. Stress.

[21] Zhao G, Zhou S, Davie A, Su Q (2012). Effects of moderate and high intensity exercise on T1/T2 balance. Exerc Immunol Rev.

[22] Moreira A, Delgado L, Moreira P, Haahtela T (2009). Does exercise increase the risk of upper respiratory tract infections?. Br Med Bull.

[23] Petibois C, Cazorla G, Poortmans JR, Deleris G (2003). Biochemical aspects of overtraining in endurance sports: the metabolism alteration process syndrome. Sports Med.

[24] Ogonovszky H, Sasvári M, Dosek A, Berkes I, Kaneko T, Tahara S (2005). The effects of moderate, strenuous, and overtraining on oxidative stress markers and DNA repair in rat liver. Canadian Journal of Applied Physiology.

[25] Hohl R, Ferraresso RL, De Oliveira RB, Lucco R, Brenzikofer R, De Macedo DV (2009). Development and characterization of an overtraining animal model. Med Sci Sports Exerc.

[26] Petersen AM, Pedersen BK (2005). The anti-inflammatory effect of exercise. J Appl Physiol.

[27] Wallberg L, Mikael Mattsson C, Enqvist JK, Ekblom B (2011). Plasma IL-6 concentration during ultra-endurance exercise. Eur J Appl Physiol.

[28] Kim H, Shin MS, Kim SS, Lim BV, Kim HB, Kim YP (2003). Modulation of immune responses by treadmill exercise in Sprague-Dawley rats. J Sports Med Phys Fitness.

[29] Lira FS, Rosa JC, Pimentel GD, Tarini VA, Arida RM, Faloppa F (2010). Inflammation and adipose tissue: effects of progressive load training in rats. Lipids Health Dis.

[30] Halson SL, Jeukendrup AE (2004). Does overtraining exist? An analysis of overreaching and overtraining research. Sports Med.

[31] Ostrowski K, Rohde T, Zacho M, Asp S, Pedersen BK (1998). Evidence that interleukin-6 is produced in human skeletal muscle during prolonged running. J Physiol.

[32] Ostrowski K, Hermann C, Bangash A, Schjerling P, Nielsen JN, Pedersen BK (1998). A trauma-like elevation of plasma cytokines in humans in response to treadmill running. J Physiol.

[33] Gleeson M (2007). Immune function in sport and exercise. J Appl Physiol.

[34] Bijeh N, Nazem F, Tavakol Afshari J, Nejat Shokouhi A, Mahmoudi M, Rastin M (2000). The effect of eccentric exercise patterns on immune system parameters of female athletes. Iran J Basic Med Sci.

[35] Pedersen BK, Febbraio MA (2008). Muscle as an endocrine organ: focus on muscle-derived interleukin-6. Physiol Rev.

[36] Febbraio MA, Steensberg A, Keller C, Starkie RL, Nielsen HB, Krustrup P (2003). Glucose ingestion attenuates interleukin-6 release from contracting skeletal muscle in humans. J Physiol.

[37] Jonsdottir IH, Schjerling P, Ostrowski K, Asp S, Richter EA, Pedersen BK (2000). Muscle contractions induce interleukin-6 mRNA production in rat skeletal muscles. J Physiol.

[38] Lancaster GI, Gleeson M (2006). Chapter 10 - Exercise and cytokines. Immune Function in Sport and Exercise.

[39] Keller C, Steensberg A, Pilegaard H, Osada T, Saltin B, Pedersen BK (2001). Transcriptional activation of the IL-6 gene in human contracting skeletal muscle: influence of muscle glycogen content. FASEB J.

[40] Keller C, Keller P, Marshal S, Pedersen BK (2003). IL-6 gene expression in human adipose tissue in response to exercise--effect of carbohydrate ingestion. J Physiol.

[41] Steensberg A, van Hall G, Osada T, Sacchetti M, Saltin B, Klarlund Pedersen B (2000). Production of interleukin-6 in contracting human skeletal muscles can account for the exercise-induced increase in plasma interleukin-6. J Physiol.

[42] Pedersen BK, Steensberg A, Keller P, Keller C, Fischer C, Hiscock N (2003). Muscle-derived interleukin-6: lipolytic, anti-inflammatory and immune regulatory effects. Pflugers Arch.

[43] Febbraio MA, Hiscock N, Sacchetti M, Fischer CP, Pedersen BK (2004). Interleukin-6 is a novel factor mediating glucose homeostasis during skeletal muscle contraction. Diabetes.

[44] Brandt C, Jakobsen AH, Adser H, Olesen J, Iversen N, Kristensen JM (2012). IL-6 regulates exercise and training-induced adaptations in subcutaneous adipose tissue in mice. Acta Physiol.

[45] Suzuki K, Yamada M, Kurakake S, Okamura N, Yamaya K, Liu Q (2000). Circulating cytokines and hormones with immunosuppressive but neutrophil-priming potentials rise after endurance exercise in humans. Eur J Appl Physiol.

[46] Steensberg A, Keller C, Starkie RL, Osada T, Febbraio MA, Pedersen BK (2002). IL-6 and TNF-alpha expression in, and release from, contracting human skeletal muscle. Am J Physiol Endocrinol Metab.

[47] Steensberg A, Fischer CP, Keller C, Moller K, Pedersen BK (2003). IL-6 enhances plasma IL-1ra, IL-10, and cortisol in humans. Am J Physiol Endocrinol Metab.

[48] Northoff H, Berg A, Weinstock C (1998). Similarities and differences of the immune response to exercise and trauma: the IFN-gamma concept. Can J Physiol Pharmacol.

[49] Lancaster GI, Halson SL, Khan Q, Drysdale P, Wallace F, Jeukendrup AE (2004). Effects of acute exhaustive exercise and chronic exercise training on type 1 and type 2 T lymphocytes. Exerc Immunol Rev.

[50] Webster JI, Tonelli L, Sternberg EM (2002). Neuroendocrine regulation of immunity. Annu Rev Immunol.

[51] McAlees JW, Smith LT, Erbe RS, Jarjoura D, Ponzio NM, Sanders VM (2011). Epigenetic regulation of beta2-adrenergic receptor expression in T(H)1 and T(H)2 cells. Brain Behav Immun.

[52] Itoh CE, Kizaki T, Hitomi Y, Hanawa T, Kamiya S, Ookawara T (2004). Down-regulation of beta2-adrenergic receptor expression by exercise training increases IL-12 production by macrophages following LPS stimulation. Biochem Biophys Res Commun.

[53] Neto JC, Lira FS, de Mello MT, Santos RV (2011). Importance of exercise immunology in health promotion. Amino Acids.

